# Exploratory research into cancer patients’ attitudes to clinical trials

**DOI:** 10.3332/ecancer.2014.432

**Published:** 2014-05-20

**Authors:** Corina W Ramers-Verhoeven, Francesco Perrone, Kathy Oliver

**Affiliations:** 1 Global Oncology Corporate Affairs, Eli Lilly and Company, Houten 3991 RA, The Netherlands; 2 Clinical Trials Unit, The National Cancer Institute of Naples, Naples 80131, Italy; 3 International Brain Tumour Alliance (IBTA), Tadworth, Surrey KT20 5WQ, United Kingdom

**Keywords:** cancer clinical trials, qualitative survey, patient participation, patient attitudes, awareness, perception, cancer, research, PACE, patient access, continuous innovation

## Abstract

A qualitative survey was carried out in six countries to gather insights into potential barriers to patient participation in cancer clinical trials (CTs) to help stakeholders develop strategies to improve recruitment and participation. While the research was exploratory in nature, the findings highlight the critical role that doctors play in terms of CTs participation. The research also indicates the need for outreach to raise awareness about CTs both outside and within the clinical research community as well as educating the general public to dispel misconceptions about CTs. The results also indicated that most patients who participated in the research believe all patients should be offered the chance to participate in CTs, wanting all available options presented to them.

## Introduction

Cancer innovation has experienced tremendous progress since the US National Cancer Act of 1971, an event that marked what has become known as the beginning of the War on Cancer. Since then, advances, including the development of more effective radiation therapy, more precise surgical procedures, and tailored targeted therapies, have—for some cancers—dramatically improved patient longevity while reducing side effects. As a result of this continuous scientific innovation, the lives of millions of cancer patients have been transformed, with cancer death rates falling for the first time in the 1990s [1]. Nevertheless, the need for continued innovation and progress in many different types of cancer remains compelling, clear, and urgent.

As an essential tool for the evaluation of medical technologies and to maintain the pace of cancer innovation, clinical trials (CTs) lie at the heart of future progress and the continued fight against cancer. Unfortunately, one of the major stumbling blocks to progress is that the number of cancer patients enrolling in trials is extremely low. This adds considerably to the time it takes to develop innovative therapies as well as increasing the costs involved.

Whereas approximately 60–70% of paediatric patients (age ≤15 years) participate in trials, the numbers decrease dramatically with increasing age, such that as few as 2–4% of all newly diagnosed adult US cancer patients participate [2, 3]. In addition, or perhaps due to low patient participation rates, a substantial number of phase III oncology trials, up to 45% in some cases, are terminated due to insufficient accrual to meet scientific objectives [4].

In 2012, Lilly Oncology formed a global network dedicated to encouraging policy changes that accelerate research and development in the cancer field and improve patient access to the most effective treatments. This network is called patient access to cancer care excellence (PACE). Operating under a transparent action plan, PACE includes stakeholders from diverse sectors involved in cancer research and development (R&D), care delivery, policy, and patient advocacy [5].

As a first step, PACE commissioned a rigorous opinion-research project focused on the general public as well as cancer patients and caregivers to establish a baseline of cancer knowledge and attitudes in six countries, the PACE cancer perceptions index. The results showed that, while most respondents’ recognised that progress has been made in cancer treatment and a majority express satisfaction with this progress, they nevertheless want more investment and faster access to new cancer treatments [6]. Moreover, the public does recognise CTs as an opportunity to advance medical research and receive better treatments not currently available. Nearly three-quarters of public respondents claimed they would be willing to participate in CTs if they were a patient themselves and it would either improve hope of receiving a life-extending treatment or improve the likelihood of helping future patients (74% and 72%, respectively).

However, this expressed willingness is very different from the reality.

As a follow-on step, PACE commissioned a qualitative research survey amongst cancer patients and caregivers in six countries to explore potential barriers to patient participation in cancer CTs and help stakeholders develop strategies to improve recruitment and participation. The findings are presented in this short communication.

## Methods

The primary aim of the research was to gain insight into cancer patients’ attitudes to CTs based on their own experience and discussion. As such, the research took the form of exploratory, qualitative interviews undertaken in six countries: the United States, the United Kingdom, Germany, France, Italy, and Japan. In total, 20 in-depth interviews were carried out in each country—eight with cancer patients and 12 with caregivers of cancer patients. Patients were recruited to achieve a mix of tumour types, cancer stages, and grades as well as experience with CTs.

Overall, 48 patients with cancer and 72 caregivers of cancer patients took part in the research. Of the patients, seven had taken part in a CT, nine had discussed CTs with their doctor but not taken part, and 32 had not discussed/taken part in a CT. Patients had a mix of tumour types. In the caregiver group, five of the patients for whom they cared had taken part in a CT, 14 had discussed but not taken part in a CT, and 53 had neither discussed nor taken part in a CT. Again, the patients had a mix of tumour types.

All interviews were conducted by telephone, with the exception of Japan, where interviewing was done face-to-face in respondents’ homes. Patient interviews lasted approximately 30 min, while the caregiver interviews lasted approximately 45 min.

Given the study’s serious and somewhat sensitive subject matter and consideration that participants may not have been feeling their best at the time of the interview, the researchers chose to implement a unique design that allowed them to access the patient voice both through the patients themselves and through their caregivers. Before their own interviews, the caregivers were requested to have a one-on-one guided discussion with the person they care for and to complete some notes that were then given to the moderator for additional context and follow up probes. This approach enhanced the depth and quality of the information derived from direct interview. It also had the somewhat unintended benefit of bringing the patient and caregiver closer together as they discussed the cancer journey of the patient. Recruitment and fieldwork took place in July and August 2013 through a mixture of patient databases and flyers placed in physicians’ offices.

This paper is qualitative in nature and intended to provide insights and direction rather than precise assessments. The findings are based on a very small sample of specifically recruited respondents. As such, they represent the opinions and attitudes of only the respondents who were interviewed. Words such as ‘some’, ‘most’, and ‘few’ categorise the findings from the study participants and are not representative of the general public. Quotes from caregivers come from their interviews with patients and are intended to reflect the experience and opinions of the patient for whom they care.

## Results

Most respondents said their doctors did not bring up the option of taking part in a CT during the treatment-planning phase. This is consistent with the quantitative results from the PACE cancer perceptions index indicating that 73% of patients did not recall discussing CT participation with their doctor [6]—slightly lower than an earlier survey of 6000 cancer patients which found that 80% were unaware that CT enrolment was a treatment option [7].

Not surprisingly, CTs were not top-of-mind for individuals when they were diagnosed with cancer. Not only was awareness of CTs low amongst the cancer patients, but—when, during the course of this research, the concept of CTs was raised—most tended to believe that if participation was suitable or best for them their doctors would have discussed it with them.

‘I didn’t ask my doctor about participating in a clinical trial because it never occurred to me’ (UK respondent/patient).

‘I would have expected the hospital to ask if I was interested in taking part in clinical trials if there were any. Having been given the diagnosis of cancer, there were many other things that I wanted to ask and I don’t think clinical trials crossed my mind’ (UK respondent/patient).

‘As the doctor never talked to me about clinical trials, I would not ask people about how to participate in one’ (Japanese respondent/patient).

Overall, there was a feeling that when conversations about CTs did take place, the conversations were more of a one-sided directive rather than a dialogue between the involved parties.

‘The oncologist saw my age and asked himself the question of a clinical trial for me…He was thinking aloud with his junior doctor. He was wondering if I was eligible. But, actually, I wasn’t eligible. Everything went very quickly. It took under two minutes…he asked: what are the conditions to enrol in this clinical trial? And was told it was under 40 years. As I was 40, I wasn’t eligible’ (French respondent/patient).

Moreover, in the minority of cases, when patients did raise the question of CTs themselves with their doctor, some felt that their doctors were somewhat dismissive of the concept or only hastily addressed their questions, leaving patients puzzled and even frightened.

Interestingly and perhaps not surprisingly, attitudes to CTs can change over time as people come to terms with the shock of a diagnosis and may become more proactive in terms of their care.

‘I became aware of clinical trials later. At the beginning, you are involved emotionally, so you trust and base yourself on what your physician tells you. Later, when my mind was clearer, I started realising that there were also these trials’ (Italian respondent/caregiver of patient who did not participate in CTs).

The spontaneous associations of CTs among patients were mixed. One German caregiver whose husband did participate in a trial said that, for her, it was first and foremost about hope:

‘The hope that there is a new medication in which my husband and patients in general can participate, hopes that the cancer will be stopped, that research can possibly manage to find something with which this disease will be treated. You think about hope rather than risks and side effects’ (German respondent/caregiver).

By contrast, others saw CTs as a form of hopelessness, like this French patient:

‘I think they are for when all hope is gone and that there is no other alternative. The patients who participate in a trial really have no other choice’ (French respondent/patient).

There is also a concern about the unknown and the perception of being a guinea pig:

‘There is always doubt, since it is a proposal for investigation. To investigate means to be a guinea pig, and this means the outcome could be positive or negative’ (Italian respondent/caregiver).

‘When the oncologist saw that the treatment wasn’t working, they told him they could try something else. But they presented the thing as if he were a guinea pig’ (French respondent/caregiver of a patient who did not take part in the CT).

### Misconceptions

The research also suggested some important misconceptions about how oncology CTs works, in particular, terms of understanding of the placebo. The most common belief among the patients interviewed was that receiving a placebo meant that the patient would not be getting any cancer therapy at all. Not surprisingly, this was regarded as a major barrier to participation, making CTs seem risky and unsafe.

‘It is always at the back of your mind that you might be in the placebo group and think I am not taking anything for months because I am not in the right group’ (French respondent/patient).

‘I would never even consider it…I don’t want to play around with medicine and risk getting the placebo’ (US respondent/patient).

Only a minority—usually those who had gone through a CT—understood that cancer patients always receive standard care regardless of whether they are in the investigational or placebo treatment group in a CT. This is similar to the PACE cancer perceptions index findings where 43% of the public were under the misconception that some patients with cancer taking part in CTs would receive only placebo and not active treatment in addition to the placebo [6]. This is something that needs to be addressed as a matter of priority both within the oncology clinic and more widely among the public.

When it comes to who should participate in CTs, most respondents in the qualitative survey viewed CTs as relevant for advanced-stage patients only, where standard treatments had failed, as expressed by this UK patient:

‘I thought that clinical trials were only for a last resort when you have nothing to lose’ (UK respondent/ patient).

Another US patient who did not participate in a trial said:

‘I get the impression that a clinical trial is one of these last-step things, which may not be true, but that’s from what I‘ve seen’ (US respondent/patient).

That said, in the context of the research discussion, some patients did make a case for why less-advanced-stage patients might be able to benefit, even suggesting that a treatment under investigation might actually work better and faster for patients at an earlier and, therefore, healthier stage. Some also voiced the idea that young patients might be better equipped to handle possible side effects and that such patients might have more to live for.

The benefits of CTs were recognised by the survey group: helping to advance medicine and treatment outcomes, having better treatments with fewer side effects, and helping future patients were cited time and time again. However, there was a disconnect between this view and the view that participation is ultimately not for the benefit of the individual’s situation, and this could lead to feelings of fear, risk, and uncertainty for the patient.

Nevertheless, once information about CTs was presented and discussed in the course of the research interview, there was almost universal agreement that all cancer patients, regardless of age, life, stage, or severity of disease, should at least be offered the chance to participate in a CT. The research subjects also almost universally agreed that they wanted to be armed with information about all available options to allow them to make an informed choice along with their doctor.

‘Everyone should be given the same chance to get new treatments or advanced treatment regardless of whether they are in an early or late stage’ (Italian respondent/patient who did participate in a trial).

‘Clinical trials should be offered along with other treatment options…the patient should know everything they have to choose from’ (US respondent/caregiver).

### Participant experience

Overall, and for those patients who had participated in a CT, the experience was generally regarded as very positive throughout the duration of the trial.

‘I don’t think there were any disadvantages; maybe it was hard for my uncle to go for a visit or for one or more tests when he was tired or had therapy-related side effects, but actually there were no disadvantages’ (Italian respondent/caregiver).

Indeed, most CT participants praised their experience on a trial, stating participation made them feel special and part of a privileged group who received better and more frequent monitoring by doctors and other health-care professionals.

‘He [the patient] was monitored completely differently than the other cancer patients on his ward. Yes, that was notable simply down to the way he was cared for’ (German respondent/caregiver).

‘He [the patient] would recommend clinical trial participation to others because he feels he is part of an almost privileged group, stating he had access to special therapies, and there are more and closer controls than for standard patients’ (Italian respondent/caregiver).

Unfortunately, this feeling of being special in many cases vanished when participation was over. Although all participants were appreciative of the care they received during the trial, there was a very clear sense of feeling that they were no longer a priority when the trial ended.

‘You are extremely well informed, but once you come off the trial there is not one letter. Nothing...This is the major problem I had with it’ (French respondent/patient).

Many also expressed frustration at never being told the results of the CT in which they participated:

‘The clinical trial experience was similar to how I had imagined it, but I was surprised that I didn’t get more information about it all as it progressed and when I was withdrawn’ (UK respondent/patient).

‘Will the results of the clinical trial be provided? That’s what preoccupied me the most’ (Japanese respondent/patient).

## Discussion

While qualitative studies do not aim at claims about the entire population, the findings here suggest possible patterns that may exist among the patient population in their attitudes to CTs and about some of the reasons behind the low levels of participation in such trials.

First, the findings reinforce the belief that awareness of CTs among patients remains an issue and that patients and the public would benefit from education that dispels some of the myths that are clearly held and informs them about what motivates CTs, how they are organised, and their potential benefits to individuals and society.

The findings also support the view that patients’ doctors hold the key to CT participation and effectively act as the gatekeepers to patients even considering participation. Reassuringly, the patients involved in this research generally seem to have high levels of trust and confidence in oncologists, as evidenced by the fact that most patients expressed the belief that doctors would mention CTs if that were the best treatment option for them. Yet, the findings of this research are consistent with previous research, indicating that many oncology physicians are currently not discussing CTs with cancer patients. It would be of interest to investigate this further, from the perspective of oncologists. Even so, going forward there would appear to be a need to consider ways to support health-care professionals in addressing CT participation with their patients to enhance quality discussions and informed decision making.

Interestingly, there was no doubt that most of the patients involved in this research, once they understood more about cancer CTs as a result of the dialogue with the researchers, did want choice when it comes to CTs and did think that all patients should be offered the chance of participating in CTs to ensure that everyone was able to benefit from all available options presented to them.This shows that patients are open to receiving information and to discussions about CTs. This is consistent with the results from our previous research indicating that 73% of respondents think patients should have the opportunity to participate in CTs.

However, greater discussion of CTs requires that CT information reaches all doctors treating cancer and that those doctors are motivated to discuss CTs with patients at the treatment planning phase. Consideration could, for example, be given to making the discussion of CT options an essential part of the standard of care for the treatment of cancer patients. Hand-in-hand with this could be the expansion of training programmes on the conduct of CTs for oncologists, to enhance their familiarity and comfort with the logistics of CTs. Of course, making discussion of CT participation part of standard care needs to be balanced against ethical considerations. For instance, it is important that oncologists not only give complete information to patients but also have a neutral attitude, neither persuading nor dissuading them to participate.

Attention could also be placed on enhancing the quality and improving access to existing resources about CTs so doctors would find it easier to identify appropriate CTs for an individual patient. For example, consideration could be given to establishing a centralised patient registry, the sort of which is already well established in the paediatric patient population, to improve the success with which appropriate patients are identified for CTs.

Finally, the CT participants interviewed in this research were generally very positive about their experience, and, interestingly, these individuals suggested that they could act as important role models for others to help cancer patients considering participation. It would seem likely that patient role models who have been part of CTs could play a valuable role in educating future patients to allow them to make an informed choice about CT participation.

In short, continued development of cancer therapeutics requires well-designed CTs, which must include a critical mass of representative, dedicated, willing, and fully informed participants. Given that the rate of CT enrollment among all cancer patients is less than 5%, the existing CT infrastructure is clearly insufficient. The urgency surrounding this issue is particularly pressing given anticipated changes in the fundamental structure of cancer CTs as the emphasis begins to shift from large-scale studies of relatively unselected patients to smaller studies testing more narrowly targeted therapies in molecularly characterised populations.

CTs will become smaller. However, smaller trials may not necessarily move faster or be easier to complete, as they will require the right patients, who will be harder to find. In particular, treatments that target relatively rare mutations will require a large number of potential volunteers to be screened to identify sufficient numbers with the relevant target.

Clearly, the issue of patient enrollment in cancer CTs warrants urgent action, and while qualitative studies are not designed to make claims about the entire population, the findings here suggest possible patterns that may exist among the patient population, in particular about some of the reasons behind the low levels of participation in CTs. We remain open to the prospect of conducting a larger quantitative study of patients and caregivers to validate the patterns uncovered in this exploratory qualitative research project for a more effective interpretation and justification of the results. It would, for example, be interesting to find out more about differences between the experiences of patients who are seen by primary care physicians and research oncologists, a distinction not make in this particular study. It would also be interesting to find out more from doctors’ perspective about the ethical dilemmas they face in discussing CT participation with patients particularly advanced cancer patients who might be prone to accept any options.

### Summary of suggested actions

- Ensure CT information reaches all oncologists.
- Motivate clinicians to discuss CTs with patients at the treatment planning phase.
- Make discussion of CTs options part of standard care in oncology.
- Expand oncologists’ training programmes on the conduct of CTs.
- Enhance quality and access to existing resources about CTs for both doctor and patient.
- Establish a centralised patient registry to assist in the recruitment for CTs, especially for rare cancers.
- Develop CT ‘ambassadors’, patients who have already participated in a CT and can tell others what it is like in terms of pros and cons that are realistic and stem from real-world experience.

## References

[1] American Cancer Society (2014). Cancer Treatment & Survivorship Facts & Figures 2012-13.

[2] Lara PN (2001). Prospective evaluation of cancer clinical trial accrual patterns: identifying potential barriers to enrollment. J Clin Oncol.

[3] Swain-Cabriales S (2013). Enrollment onto breast cancer therapeutic clinical trials; A tertiary cancer centre experience. Appl Nurs Res.

[4] Schroen AT (2010). Preliminary evaluation of factors associated with premature trial closure and feasibility of accrual benchmarks in phase III oncology trials. Clin Trials.

[5] www.PACENetwork.com.

[6] Ramers-Verhoeven C (2013). New insights into public perceptions of cancer. ecancer.

[7] Comis RT (2000). A quantitative survey of public attitudes towards cancer clinical trials. Proc Am Soc Clin Oncol.

